# Beyond individual factors: a critical ethnographic account of urban residential fire risks, experiences, and responses in single-room occupancy (SRO) housing

**DOI:** 10.1186/s12889-024-19866-z

**Published:** 2024-08-28

**Authors:** Catherine Liao, Colleen Varcoe, Helen Brown, Ian Pike

**Affiliations:** 1 School of Nursing, Faculty of Applied Science, University of British Columbia, Westbrook Mall, Vancouver, BC 2211, V6T 2B5 Canada; 2 Department of Pediatrics, Faculty of Medicine, University of British Columbia, BC Children’s Hospital, Rm 2D19, 4480 Oak Street, Vancouver, BC V6H 3V4 Canada; 3 BC Injury Research and Prevention Unit, 4500 Oak Street, Vancouver, BC V6H 3V4 Canada

**Keywords:** Single-room occupancy, Inadequate housing, Fire risks, Burn injuries, Underserved communities, Ethnographic design, Participant observation, Structural factors, Inequities

## Abstract

**Background:**

Burn injuries are a significant public health concern, closely linked to housing conditions and socioeconomic status. Residents in socioeconomically deprived neighbourhoods are at increased risk of exposure to hazards due to older and poorer housing conditions and limited access to fire protection measures. Individual behaviours such as substance use, smoking, and hoarding are often highlighted as primary causes of residential fires, overshadowing the broader socioeconomic and structural factors that also play a significant role in housing safety. This paper explores the correlation between inadequate housing conditions and heightened fire risks leading to burn injuries, focusing on the contextual factors shaping everyday urban fire risks, experiences, and responses of residents living in Single-Room Occupancy (SRO) housing in Vancouver’s Downtown East Side (DTES) and staff working in the fire, health, housing (social and private), and non-profit sectors.

**Methods:**

As part of an ongoing ethnographic study, we partnered with the Vancouver Fire Rescue Services (VFRS) to conduct participant observations in private, non-profit, and government-owned SROs, modular homes, and a temporary shelter. This paper synthesizes insights from participant observations from the first author’s self-reflexive journals, including informal conversations with approximately fifty-nine individuals such as SRO tenants, SRO managers/caretakers, health workers, burn survivors, municipal staff, not-for-profit staff, and firefighters.

**Results:**

Urgent housing-related issues contributing to inequitable everyday urban fire risks were identified, such as structural deficiencies in SRO buildings and systems, inadequate waste management and storage, and inequitable approaches to addressing hoarding. Additionally, disparities in access to information and the interaction between interpersonal and structural stigmas were significant factors, underscoring the pressing need for intervention.

**Conclusion:**

Communities like DTES, facing precarious housing conditions, disadvantaged neighbourhoods, and complex health and social challenges, necessitate a comprehensive and holistic approach to fire prevention and safety. Recognizing the interplay between housing instability, mental and physical health issues, unregulated toxic drug supply, drug criminalization, and structural inequities allows practitioners from various sectors to develop contextually driven fire prevention strategies. This multifaceted approach transcends individual-level behaviour change and is crucial for addressing the complex issues contributing to fire risks in underserved communities.

**Supplementary Information:**

The online version contains supplementary material available at 10.1186/s12889-024-19866-z.

## Introduction

Housing, a critical social determinant of health, is central to individuals’ well-being and ability to thrive [1, 2]. Unstable housing contributes to premature morbidity and mortality [3]. International human rights law acknowledges peoples’ rights to an adequate standard of living, encompassing adequate housing beyond four walls and a roof [4]. Nation States are obligated to protect and promote human rights, including the right to good health and well-being (sustainable development goal #3) and sustainable cities and communities through adequate housing conditions (sustainable development goal #11) for all individuals [4, 5].

Adequate housing must meet several fundamental criteria, including security of tenure, affordability, habitability, accessibility, location, availability of services, materials, facilities, infrastructure, and cultural adequacy [6]. Structural deficiencies, insufficient insulation, lack of proper heating and ventilation, inadequate facilities for food storage and preparation, limited access to Water, Sanitation and Hygiene (WASH) facilities, presence of household pests such as cockroaches, mice, and rats, exposure to noise pollution, and risk of asbestos exposure are some examples of inadequate housing conditions [7, 8]. Poor housing conditions are closely linked to health impacts such as respiratory illness, asthma, lead poisoning, mental health, and injuries, including burns [9]. Indigenous Peoples, ethnic minorities, and people living with low incomes face compounding disadvantages exacerbated by inadequate housing conditions [8] and their physical, economic, and social realities [10].

Increasing income inequality and housing insecurity have led to more people experiencing homelessness and living in urban encampments, with anti-homeless policing and restrictive housing provisions further limiting their ability to express a sense of home [11]. A person’s sense of place, or “home,” can foster a sense of belonging and comfort, significantly impacting their well-being. Thus, homes possess significant social, psychological, and emotional dimensions for individuals and groups, extending beyond merely the physical structure of a house [10]. Homelessness is not just a matter of lacking shelter but involves deprivations in physiological, emotional, privacy, identity, and familiarity needs, all of which impact an individual’s well-being [12]. Additionally, contextual factors such as previous positive tenancy experiences, tenants’ perceptions of housing quality, neighbourhood quality and social support within the area are crucial in understanding the relationship between housing and individuals’ well-being [13]. Therefore, when considering an individual’s well-being and ability to thrive, adequate housing^1^ must be understood as a multidimensional phenomenon [14].

## Background: inadequate housing – a Catalyst for burn injuries and fire hazards

Burn injuries exemplify one facet of the inequitable impacts of inadequate housing on health. Socioeconomic deprivation and neighbourhoods with higher Area Deprivation Index are closely associated with an elevated risk of burn injuries [15–17], with residential fires being the leading cause of burn-related injuries and fatalities [8, 16]. Individuals residing in deprived areas face heightened exposure to hazards due to the prevalence of older and poorer housing conditions and limited access to fire protection measures [18, 19]. This exposure includes living in low-income housing that is outdated, poorly refurbished, and inadequately maintained [20]. For example, temporary accommodations provided to individuals experiencing homelessness pose significant fire hazards due to the structural condition and design of these accommodations [9]. Properties that are rented, municipality-owned, or managed by social housing associations have been identified in the literature as key risk factors for unintentional house fire incidents, injuries, and deaths [21]. Such harmful physical exposures are exacerbated due to unequal power relationships, especially when tenants fear eviction and thus do not complain of poor conditions because of socioeconomic disadvantage [22].

Individuals residing in deprived areas are also more likely to experience poorer access to essential burn care and follow-up services [18], with unhoused populations being more susceptible to burn injuries [23]. Their burden of burn injuries is further exacerbated by the existing co-morbid struggles with mental health issues in the context of substance use [23, 24] and various physical and emotional trauma experiences. In addition, tenants with mental health challenges living in supported housing face excessive house rules and fire safety measures that can inadvertently lead to feelings of marginalization and othering [25].

Residential fires and burn injuries have been linked to behaviours such as smoking, alcohol consumption, and substance use [26–28], and smoking materials are cited as the leading cause of death [29]. Additionally, there has been a lack of comprehensive analysis to investigate the relationships between fire incidents in impoverished neighbourhoods [29] and the impacts of decades of deregulation, privatization, and austerity measures within public services [30]. These factors contribute to affordability challenges, overcrowding, and homelessness, exacerbating the risk of residential fires in these communities, as seen in the tragic Grenfell Tower fire in London, UK^2^, and the Winters SRO hotel fire in Vancouver^3^. These two examples of the worst residential fire disasters in wealthy nations such as the UK and Canada serve as a reminder of the lived effects of austerity measures within a neoliberal agenda. People experiencing poverty are subjected to stigmatizing narratives that legitimize and normalize disparities, with poverty and economic disadvantage presented as the result of individual behaviour instead of punitive policies and political decisions [33]. Thus, focusing on individual-level factors obscures the broader contextual factors as to why certain populations face disproportionate fire incidents and burn injury risks.

## The canadian context: single room occupancy (SRO) housing and residential fires

As affordable housing becomes increasingly scarce, individuals experiencing material and social disadvantage are compelled to seek refuge in Single Room Occupancy (SRO) housing in disadvantaged communities in North America [34]. These SRO units typically consist of rooms 100 square feet or smaller, sparsely furnished with limited cooking facilities and communal bathrooms [34]. Some SROs may provide a hot plate in the room for tenants to prepare warm meals, while others lack cooking facilities. SRO housing is often the last resort for individuals before they are forced into homelessness [35].

Since 2007, the British Columbia (BC) government has been acquiring and leasing SRO hotel buildings in the DTES and surrounding areas to maintain affordable housing options for low-income individuals and those at risk for homelessness [36]. There are 146 SRO buildings with approximately 6,567 rooms in Vancouver, with 48% of the SROs privately owned, 11% by non-profit organizations, 32% by BC Housing (Crown Corporation of the BC Government), 7% by the City of Vancouver, and 2% by the Chinese Benevolent Society [37]. Even though the City of Vancouver recognizes SROs as outdated, unaffordable, and unsanitary, SROs are still being used to support individuals transitioning to stable housing [38]. Non-profit-managed SROs offer additional community support services to facilitate transitions. The BC provincial government has funded building renovations, including seismic upgrades, heritage building rehabilitation, and fire safety improvements [38].

The government has faced criticism for using SROs as a housing option due to their poor maintenance and unsanitary conditions, triggering a public health crisis [39, 40]. Living in substandard SROs in Vancouver’s DTES is clearly linked to physical and mental illness, poverty, substance dependence, and social vulnerability [41]. Therefore, SRO accommodation illustrates the detrimental effects on health and housing, exacerbated by physical consequences such as lack of space, poor heating, ventilation, and sanitation, as well as psychosocial implications stemming from a significant lack of agency and control over one’s living environment [42]. SROs are often the only remaining housing option for low-income groups, who face concurrent oppression through dehumanization and criminalization [35]; SROs have been labelled a policy failure by housing advocates [40].

Residential and outdoor fires in the DTES neighbourhood are rising, adding to the complex health and social realities of people living in SROs. The Vancouver Fire and Rescue Services (VFRS) reported 4,309 fires, five fire-related fatalities, and 72 injuries in 2023, with increasing risks of fires observed in SRO buildings [43]. Burns unit staff within the city have observed anecdotally that there is a higher frequency of burn cases among individuals residing in poor physical environments. This includes individuals facing mental health challenges, substance use, and drug toxicity.

Burn injuries and fire events are frequently perceived as the consequence of individual behaviour rather than acknowledged as part of a broader population phenomenon. Addressing fire challenges in neighbourhoods with inadequate housing and underserved populations requires understanding how human behaviour, sociocultural, historical, sociopolitical, and socioeconomic factors and the built environment contribute to heightened fire risks. Understanding these contextual factors is crucial for developing targeted interventions to promote fire safety in the most affected communities. Focusing on fire hazards and risks solely at the individual level obscures the broader social factors and their role in perpetuating inequities within structures and systems. The findings in this paper are part of an ongoing ethnographic research project that examines the contextual factors shaping inequities in fire risks and burn injuries among underserved populations. Specifically, this paper demonstrates how the physical and social aspects of housing conditions exacerbate everyday urban fire risks for people living in SROs, using Vancouver’s DTES neighbourhood as an example.

This study was conducted on the unceded traditional territories of the xʷməθkʷəy̓əm (Musqueam), Sḵwx̱wú7mesh (Squamish), and səlilwətaɬ (Tsleil-Waututh) Nations – Vancouver. Most SRO housing in Vancouver, Canada, is concentrated in densely populated urban areas such as the DTES [41]. The DTES is one of the oldest neighbourhoods in the heart of Vancouver [44] and is characterized by its cultural diversity, with 48% of its population representing visible minority groups^4^. The community includes residents from Chinatown, many First Nations people from various parts of the Americas, and numerous newcomers to Canada. The DTES has a higher proportion of seniors, a significant number of low-income families, and single individuals who are unemployed or experience prolonged periods of unemployment [4^4^, 46].People experiencing long-standing homelessness and severe psychiatric comorbidities have migrated to the DTES neighbourhood from other areas due to the high concentration of services available here [47].

### Methods

#### Qualitative approach and research paradigm

The findings in this paper are part of an ongoing ethnographic design research project. As Hammersley and Atkinson [48] described, ethnography is a methodological approach adapted and reinterpreted across different disciplines to suit evolving circumstances. Despite its varied interpretations, ethnography aims to engage, interpret, and document social phenomena within everyday contexts [49]. Critical ethnography (CE) distinguishes itself by its commitment to uncovering hidden agendas, challenging taken-for-granted assumptions, fostering conditions for greater equity and being guided by an ethical imperative to disrupt the status quo [50].

Ethnography often relies on data gathered through observing daily practices and interactions to understand everyday contexts effectively, including examining elements like signage and physical environments and analyzing documents such as policies and directives. In this case, participant observation included observing the daily activities of fire inspectors, SRO tenants, and staff, supported by data from fire reports and publicly available meeting memos (municipal) and policy documents. We examined fire risks and burn injuries through the lens of social justice, human rights, and equity, thereby reinforcing a public health perspective that extends beyond individual behaviours [51]. The study received approval from the University of British Columbia’s Behavioural Research Ethics Board and the First Nations Health Authority^5^, BC (H22-03219).

### Researcher characteristics and reflexivity

The first author is a doctoral student with over twenty years of nursing experience. She is also the director of a registered Scottish Charity dedicated to enhancing health systems in burn care in low-resource settings in West Africa. To ensure reflexivity, the first author critically analyzed how researcher positionality, subjectivity, and context influenced the research process.

The first author also maintained a series of analytic memos, asking specific questions, interpreting responses, and documenting observations in field notes. This method facilitated a critical examination of the first author’s judgments, practices, and belief systems during participant observation [52].

This research was guided by CE methods, prioritizing, understanding and considering power dynamics and contextual influences, recognizing that reality is socially constructed and subjective [49]. The first author engaged with the co-authors (supervisor and committee members) throughout the research process, seeking guidance on ongoing data collection, analysis, and the writing process, including dilemmas encountered during fieldwork. The first author also continuously discussed and shared the analysis with the Assistant Chief, the VFRS team, and a few SRO staff throughout participant observation, including providing updates on the research activities.

### Data collection

In the initial stages of her doctoral studies, the first author presented the research study to the Fire Chief/General Manager and assistant fire chiefs for the VFRS. The VFRS team endorsed the project due to its relevance amidst increasing fire calls in the DTES area. The Assistant Chief of the VFRS Community Safety Division invited the first author to collaborate with the department’s Urban Compliance Team (UCT Team). The AC believed that participating in “ride-along” (shadowing the team on duty) would facilitate understanding the everyday urban fire risks in the DTES. These observations aimed to illuminate how residents of SROs and individuals working in this environment perceive and address these challenges.

The first author presented the study objectives and the consent forms to the VFRS staff. She obtained informed consent from the team members she shadowed. The first author was required to wear a visible organization identification name card. The UCT team consistently introduced the researcher as a doctoral student, shadowing them for a research project. During each field visit, she introduced herself to the tenants and staff at the various sites. She explained the purpose, the types of observations she was conducting, and the documentation she was recording. Individuals who interacted with the VFRS team in the presence of the first author provided verbal consent for her to be present during these interactions. In some instances, she was not allowed to attend certain meetings. Anonymity was guaranteed to all participants.

The first author joined the UCT Team in DTES, where the team conducted inspections to enforce Vancouver’s Fire Bylaws, encompassing SROs. The captain assigned the first author to a roster, allowing them to shadow two distinct crews during morning and afternoon shifts. The AC facilitated further opportunities, such as accompanying the Public Education department during a fire extinguisher training at a legal homeless encampment and supplying VFRS’s administrative data on fire events in DTES. The captain arranged an observational experience with the Hoarding Action Response Team (HART), which primarily conducts assessments to improve health and safety and assists individuals affected by compulsive hoarding outside the DTES area.

These observational experiences allowed the first author to engage with various individuals and interact with staff within privately owned, not-for-profit, and government-operated SROs in the DTES, including modular homes and other supportive housing facilities. Throughout participant observations, the first author established rapport and engaged with community members, including service providers and burn survivors, who subsequently took part in qualitative interviews. The participants highlighted relevant housing and fire safety policies and publicly available memos from the Vancouver municipality. They provided the first author additional insights into other related questions to explore within the research. This rapport fostered active participation among participants, leading to the co-production of data [53].

During participant observation, some individuals volunteered to converse with the first author, offering to share insights into the situation. The conversations and questions during participant observation were spontaneous and were not part of the pre-established interview guide. The participant observation and interview guide (qualitative interviews) developed for this study are uploaded as a supplementary file.

The participant observation occurred across 50 sites, including 43 SROs (privately owned, non-profit, and government), 2 privately rented accommodations outside the DTES area, 1 legal homeless encampment, 3 modular housing, and 1 temporary shelter. This paper draws insights from participant observations and conversations with 59 individuals in the field, including 13 VFRS staff, 2 municipal staff, 17 tenants, 5 mental health support workers, 6 caretakers, 8 managers, 3 SRO fire survivors, and 5 community partners (various not-for-profit organizations). Some sites were visited multiple times. Some caretakers and managers worked at multiple SRO sites due to ownership by the same organizations. Disaggregated data is not presented in this paper to maintain anonymity, as individuals working and living in DTES could be easily identified. The participant observation phase began in August 2023 and continues into 2024. Formal qualitative interviews with service providers, community leaders, and burn survivors are ongoing and will be analyzed and presented separately.

Observations were conducted at critical points, including inspections of SRO buildings violating Vancouver’s Fire Bylaws, hoarding inspections, issuance of notices to tenants and SRO managers, imposition of fines, and placement of fire watches. These observations aimed to collect contextual information on the roles and responsibilities of SRO tenants, management teams, housing associations, and municipal service providers. The participant observations sought to identify upstream factors that elevate the risk of fire and burn injuries among certain populations by examining social situations, power dynamics, and institutional influences.

The first author conversed with individuals during these observations, tailoring inquiries to the research objectives and specific situations. For instance, during hoarding inspections and reinspection, the first author observed interactions among fire inspectors, SRO staff, and tenants. Subsequently, the first author requested individuals to share their perspectives on hoarding, its fire risks, personal experiences, and responses to these challenges to gather diverse viewpoints. After each encounter, detailed notes were initially handwritten in a notebook and later typed and transcribed, documenting observations, conversations, and verbatim quotes whenever possible. In cases where individuals expressed concerns about potential reprisals, their wishes were respected by refraining from documenting verbatim and reassuring them of anonymity.

### Data analysis

Ethnographic data analysis is not formulaic; instead, it requires a flexible approach wherein the researcher is attuned to the research questions, the data involved, and the phenomena being investigated [53]. This approach recognizes that these elements may evolve, necessitating adaptability and openness to new insights and interpretations [48]. The first author used the six-phase^6^ of thematic analysis proposed by Braun and Clarke as a framework to provide structure and an audit trail for the data analysis [54]. The analysis was an iterative process foregrounding the importance of critically reflecting on how the researcher’s presence may have shaped the data and implications for analysis [48]. The first author used purposive sampling [55] to generate conversations and then organized the field notes in NVIVO ^QSR^ (version 14). The first author then engaged in an iterative process of deep analysis and critical reflexivity on the extensive reflexive notes with guidance from her supervisor.

## Findings

Efforts to address fire safety often take a reductionist approach, focusing on modifying individual behaviour and applying a one-size-fits-all solution. The increasing number of fire incidents in SRO buildings has led to a stigmatizing narrative, often blaming people who use drugs. This narrative assumes that smokers’ mishandling of materials like lighters, candles, and matches is the primary cause of fires, with little attention to underlying causes. Such narratives have intensified the interpersonal^7^ and structural stigmas^8^ experienced by those already marginalized. Social housing systems place individuals who use drugs and live in SROs under excessive regulation, preventing them from exercising their agency within their own “homes.” These narratives discredit and diminish an individual’s agency, leading to them often not being believed [56]. Interpersonal stigma perpetuates and reinforces structural stigma through policies, laws, and cultural norms, constraining the ability of stigmatized individuals to thrive in society [57].

The following section describes the DTES neighbourhood based on firsthand observations and explores key housing-related themes contributing to disproportionate urban fire risks in SROs. These themes include structural deficiencies in buildings and systems, inadequate waste management and storage, inequitable approaches to hoarding, disparities in access to information, and the interplay between interpersonal and structural stigmas.

### Glimpsing into the DTES: observational context

In the DTES, the sound of police, fire, and ambulance sirens is a constant backdrop. The streets are populated by diverse, uniformed personnel, including law enforcement officers, firefighters, and paramedics. Alongside them, individuals in yellow vests—comprising charity workers, municipal staff, and social workers—are commonly seen. Street nurses, harm reduction workers, and outreach workers, identifiable by the lanyards around their necks and naloxone kits in their backpacks, move purposefully through the area. Additionally, charity workers can often be seen maneuvering food wagons to distribute meals at street intersections, underscoring the community’s reliance on such vital support services.

Individuals sleep on the streets or remain in a “self-medicated” state, a term used by some tenants. Staff also noted that people used to shelter at bus stops, but this changed after the City of Vancouver removed the glass panel roofs and sidings from public bus stops, preventing them from serving as shelters. A diverse group of individuals of varying ages gathers outside SRO buildings, safe injection sites, community centers, non-profit buildings, temporary canopy shelters, and a Tim Horton coffee shop. They navigate with grocery carts or wheelchairs, often displaying untreated foot infections or struggling with chronic pain, osteomyelitis, and abnormal spine curvature.

On the second Wednesday of every month, individuals queue up in the street to collect their income assistance from credit union branches, ministry field offices, or Service BC offices. The queue is often long, and tenants reported that it could take up to half a day for their turn. People huddle together in the rain or cold, finding some relief only when the weather is dry. This event is commonly referred to by those working in the DTES as “Welfare Wednesday.” Some staff reported that a Montreal street gang, referred to as the “Montreal boys,” is selling drugs in the DTES and hanging around safe injection sites. Additionally, some claim that these gang members also reside in SRO buildings.

Inside the SRO buildings, some tenants gathered in hallways and communal areas, while others were unconscious in their rooms with doors left open. In response, SRO staff would call their names to rouse them or administer naloxone if they suspected an opioid overdose. Staff accessed tenants’ rooms using a master key or electronic access card. However, staff sometimes hesitated to administer naloxone^9^ due to fear of potential violence and aggression from the awakened individual. SRO and VFRS staff frequently carried naloxone. For example, during a visit to an SRO, a tenant displaying signs of paranoia, expressing fear of being followed, and appearing under the influence of substances was observed with a severe head injury, bleeding profusely. When this individual slumped to the floor, staff hesitated to administer naloxone due to concerns about potential violence upon reversing the effects of the opioids. Instead, they called the fire crew as first responders rather than an ambulance; the reason for this choice remained unclear. The challenges encountered by individuals grappling with mental and physical health issues, severe poverty, and unsanitary living conditions are further exacerbated by the repercussions of the toxic drug crisis^10^ and the criminalization of drug use. Since the BC province declared a public health emergency in 2016, more than 14,000 people have died due to unregulated fentanyl and other toxic drugs [60].

“Cultural adequacy” and accessibility are notably absent in social housing such as these SROs, as illustrated by a tenant (self-identified as Indigenous) who leaves his tap running in his SRO room to feel connected to the land and water. The high rate of homelessness among Indigenous Peoples in Canada, which is eight times higher than for people of other ethnicities, is directly linked to historical and ongoing colonial policies that perpetuate systemic racism, cultural oppression, and the dispossession of Indigenous lands [61].

Many tenants in these SROs self-identified as Indigenous. In line with the demographic profile, Indigenous Peoples make up 6% of BC’s population, yet they account for 40% of Vancouver’s homeless population, of whom half are unsheltered [62]. SRO staff were from “visible minority” groups, while managers were predominantly of European descent. The observational context is crucial, providing essential background to the themes discussed in the findings, particularly highlighting the inadequacies of SRO housing due to the prevalent fire hazards.

### Theme 1: the structural deficiencies of buildings and systems

Constructed in the early 1900s, many SRO buildings in the DTES are now unlivable and outdated, compromising the safety and dignity of tenants. Individuals working in the DTES refer to these SRO buildings as “problem buildings” due to their deteriorating conditions and “problematic” tenants. Many older buildings have exposed wires and pipes on the ceilings and walls. The carpeting shows wear and tear, appearing threadbare, and the elevator systems are often non-functional. Additionally, the doors and walls are riddled with numerous holes, with residents attempting to fill some with paper and cardboard.

Older SRO buildings commonly suffer from insufficient electrical sockets and malfunctioning heating and ventilation systems. The limited availability of functional sockets hinders residents from using portable electrical heaters and other devices. An elderly tenant shared, “I leave my gas stove on, and I set my cooker oven to 200 [Fahrenheit], and it keeps the place warm.” These heat sources pose a significant fire risk in multi-dwelling buildings. Tenants often do not use the carbon monoxide detectors provided by fire inspectors because they need to be plugged in. With only two electrical sockets available, prioritizing the CO2 detector over phones, heating devices, or lights is impractical. Tenants frequently use the electrical sockets in the hallway to plug in their electric burners to heat food. One tenant did so routinely because the socket in his room would “blow up” each time he tried to use it.

The lack of ventilation in older buildings poses significant challenges to airflow and cooling. During one August visit, outside temperatures reached 34 degrees Celsius, and the interior felt like a stifling sauna, thick with stale tobacco smoke. A caretaker reported that “residents open storm doors” to allow for airflow in the summer when temperatures rise. The SRO with the highest number of fire calls is an older building initially designed with two wings surrounding an open courtyard, which provided natural light to inward-facing rooms. Subsequent modifications to operate as an SRO, such as the courtyard being enclosed with a glass ceiling, increased fire risks by restricting smoke dispersal due to insufficient ventilation.

These structural conditions led to numerous fire code violations, fire calls, and instances of fire. Fire officials faced limited corrective options that could align with the challenging conditions faced by tenants. According to some fire inspectors, the UCT team regularly issued fines to SRO buildings for safety violations, but these measures still need significant improvements. For example, the building described above, which had the highest number of fire calls, received numerous fines and violation notices for its unsafe features, such as faulty storm doors and lack of ventilation due to glass ceilings in the enclosed atrium. It was also cited for needing more fire safety compliance, including delays in structural repairs, absence of fire safety signs, and inadequate exit lighting and signs. When fire inspectors issued fines, the managers and caretakers of these housing associations seemed to accept them nonchalantly.

On multiple occasions, fire inspectors instructed tenants to remove tape and plastic bags covering smoke detectors. The UCT team regularly reminded staff to mitigate fire risks by repairing holes in doors and walls, adding door numbers to rooms, installing adequate lighting at fire exits, enforcing no-smoking indoor rules, fixing broken fire storm doors or ensuring they remain shut, replacing empty fire extinguishers, minimizing clutter, and signposting the building number (address). For example, a tenant expressed anger about a “thirty-minute” delay in the fire crew’s response despite the building being opposite a fire hall. Although the address was listed as being on one street, access was via an adjacent street, which confused firefighters.

Older SRO buildings need modern fire suppression systems. Unlike newer buildings, many older SROs are equipped with local alarms that do not automatically notify fire halls during a fire incident. Consequently, residents are responsible for sounding the fire alarm and contacting the fire department by dialling 911 from a safe location. Caretakers noted that “tenants do not retain this information [dialling for help].” Additionally, fire inspectors reported that older buildings have “fire hose reels that do not fit the newer ones or the racks for standpipe and hose systems are too high to reach,” complicating fire response efforts. A firefighter further explained that many “SROs in heritage buildings don’t have sprinkler systems,” emphasizing that having an “integrated sprinkler system is a significant component in improving fire safety in the city.”

Some fire inspectors and SRO caretakers expressed the view that SRO accommodation should be phased out due to the poor conditions of the buildings. Some SRO staff, who have been in the DTES for a significant period, believed the area is gradually “undergoing gentrification.” In contrast, others expressed frustration because “it is difficult to find people to work here” [DTES] or “renovations take longer because contractors do not want to come and work.” Some shared that “they [contractors] are dragging the repairs.” Conversely, tenants welcomed the delays due to the fear of “renoviction,” a term they used to describe housing insecurities when property owners evict tenants under the guise of renovations, often leading to rent increases that impede their ability to return. One tenant commented in a private SRO, “I have lived in this building for eight years, and even though this building is not in good shape, I will not find another place at this rate.”

Staff members working in older SRO buildings faced a significant responsibility to maintain fire safety standards, often with limited resources. In extreme cases where fire risks are high due to deficiencies, buildings are placed on a “fire watch.” During a fire watch, caretakers check each of the common areas of the buildings every 15–30 min for fire hazards. The fire watch can extend for 1–3 days, and occasionally even longer, depending on the promptness of contractors in repairing the faulty fire safety equipment and alarm panels. One caretaker described feeling “punished by the fire watch duty,” as they were tasked with conducting “fire watch rounds” despite having other responsibilities. During a fire watch round with the caretaker, a woman stated that she uses “bolt locks for extra safety,” raising concerns about residents being “trapped” indoors during fire emergencies. Extra staff were not hired for fire watch duties, leaving SRO caretakers feeling “stretched.” There was a sense of confusion as to why the government would not invest in building a brand-new structure but instead allocate funds to retrofit older buildings. One staff member expressed this sentiment, likening it to “putting lipstick on a pig.”

Staff, particularly those overseeing non-profit-managed SRO buildings where tenants contend with significant mental, physical, and substance use challenges, voiced skepticism regarding maintaining fire safety compliance. One remarked, “These issues [fire incidents] will never go away.” In contrast, a fire inspector suggested that the only practical approach to minimize fire risks and increase compliance with fire safety regulations is “the enforcement method of fines.” Staff members also perceived that “outsourcing the housing of the disadvantaged [individuals relying on social housing] to these non-profit organizations that all have a vested interest in staying in business” was driven by financial motives. There was a widespread assumption among DTES workers that large non-profit organizations were profiting from BC Housing^11^.

### Theme 2: inadequate waste management and storage

Tiny living quarters and inadequate storage options in SRO buildings, compounded by structural issues due to aging and poor waste management, have raised concerns among fire inspectors. Garbage cans often obstruct fire exits, and when removed, tenants leave garbage in plastic bags outside their doors, creating fire hazards due to the accumulation of combustible items from domestic household waste. Many SRO buildings lacked such spaces compared to newer buildings with designated garbage amenity rooms.

The inadequate waste management solutions deeply frustrated staff in these older SRO buildings. Staff described mitigating fire risks from the accumulation of household waste as “impossible” because “the building is not purpose-built or renovated to accommodate garbage,” and simply removing garbage cans does not solve the problem. A caretaker expressed concerns that “removing the garbage cans would lead tenants to litter the place because they now had no place to throw their trash.” The waste management situation prompted one caretaker to “buy a wagon” to navigate the stairs, as the building lacked elevators, allowing them to carry the garbage and load it onto the wagon. Frustrated, a caretaker questioned, “What is the solution?” They noted that “removing garbage cans from the hallways resolved one issue but created another concerning garbage disposal.” When discussions reached an impasse, the UCT team often recommended “reaching out to building management” or “retraining the tenants” on garbage disposal, as their primary focus was fire safety concerns.

Tension between SRO staff and tenants arose due to the need for more storage space. Staff were perceived as “nagging” tenants to remove personal items such as wheelchairs, walkers, mattresses, equipment, and pets because these items obstructed hallways. The UCT team consistently reminded SRO staff to convey to tenants the importance of “keeping pathways clear for emergency responders.” An elderly tenant in an SRO expressed irritation when inspectors and staff advised her to organize her room to create more storage space: “Don’t infantilize me. I want to rent a storage space, but there is nowhere to go.” Another tenant became frustrated when reminded to move his items from the hallways: “I know all of these items need to be cleared, and I could do with a hand with moving items down because I am in my seventies, and there is only so much I can do.” This building had no elevator, and without adequate storage facilities, tenants in SRO buildings had limited options.

### Theme 3: inequitable approaches for addressing Hoarding

In single-room settings where individuals face extreme poverty, accumulating personal belongings and resulting clutter are considered fire hazards within SRO buildings. When SRO staff discover an excessive accumulation of items obstructing the everyday use of space, they refer the tenant for room inspection through Vancouver’s 3-1-1 hotline, a general dispatch system for municipal inquiries, complaints, and resources. SRO staff use a master key to access tenants’ rooms, which tenants perceive as an invasion of their privacy. Additionally, while SRO staff assert that they notify tenants about inspections, some tenants claim they receive less than a day’s notice. In some cases, tenants claim they were not informed.

The UCT team uses the Clutter Image Rating (CIR) scale to conduct hoarding and clutter assessments in SRO buildings. This tool comprises three sets of nine-color photographs depicting rooms (living room, bedroom, and kitchen) with varying levels of clutter, from 1 (least cluttered) to 9 (most cluttered) [64]. Despite its limited applicability in SROs, the CIR scale remains the primary assessment tool during inspections. Inspectors evaluate whether the SRO room doors can open to a 90-degree angle to facilitate emergency access for first responders. However, the assessment does not consider whether hoarding interferes with the tenant’s daily functioning.

Hoarding assessments in SRO buildings primarily focus on mitigating fire hazards rather than prioritizing tenant well-being. During one observation, the first author noted a tenant facing eviction due to hoarding, frantically rearranging her belongings in a room infested with cockroaches and rodent droppings. The tenant exhibited physical, emotional, and mental health challenges, such as difficulty moving items due to scoliosis and signs of distress, including angry outbursts and tears. Another tenant, facing mobility challenges, relied on friends to help tidy her room as she depended on a wheelchair. Tenants who fail to make significant improvements by the second reinspection are issued a “do not occupy” (DNO)^12^ notice, often leading to eviction.

There was a notable difference in how hoarding assessments were handled in non-SRO housing outside the DTES. When hoarding situations outside the DTES were reported through the 3-1-1 hotline, a team known as the Hoarding Assessment Response Team (HART), comprising a fire inspector and a healthcare worker, would conduct an initial visit. These assessments were in response to referrals from private rental properties or low-income housing cooperatives outside the DTES. The first author observed that a “trauma-informed” approach was used to assess hoarding cases, such as giving individuals more time (e.g., seven weeks) to declutter, listening to the tenants, connecting them to resources, and providing support. In contrast, tenants in SROs were given only one week. Some tenants in SROs expressed “mistrust” towards individuals in uniform because they had experienced “people coming into this building posing as fire officers and issuing ‘do not occupy’ signs on their doors to evict them.”

The first author received varied responses from healthcare workers and fire inspectors when asked to explain the different approaches to managing hoarding in the city. Some mentioned budgetary cuts, while others noted that DTES residents already had access to many services. However, no clear explanation was provided for why hoarding assessments outside the DTES received support from the Health Authority and followed a different protocol.

### **Theme 4: disparities in access to information**

Despite increasing fire incidents and emergency calls in the DTES, SRO tenants have limited access to comprehensive fire safety information. There is a noticeable absence of fire safety and prevention information for tenants within these buildings. Aside from visible signs issued by the fire department, such as notifications of fire safety violations or “do not occupy” signage on the doors, resources and educational materials are scarce. One tenant remarked, “Having more reminders on the walls would help us be more cautious and careful.” Although the VFRS provides extensive resources online, many SRO residents lack internet access. To address this gap, some SRO managers and caretakers have created homemade posters to raise awareness of fire risks, hoarding issues, and the dangers of storing E-bikes indoors. Additionally, an SRO manager expressed concern about the “lack of education for staff on fire safety.”

The combination of poor SRO building conditions, lack of ventilation, indoor smoking, and the presence of substance use paraphernalia, including butane torches, posed significant fire risks in these buildings. According to a fire inspector, although most SROs aim to maintain a smoke-free environment, and some have implemented rules to restrict or prohibit smoking, “these rules must be more effectively communicated or enforced.” One caretaker shared, “In [tenants’] minds, it is their room; they are paying for it, and they can do whatever they want.” Frustrated, repeatedly reminding tenants not to smoke in their rooms and common areas, another caretaker requested “no smoking signs with a big fire department stamp” from the fire inspector, believing that signage from authoritative sources would carry more weight.

### Theme 5: the interplay between interpersonal and structural stigmas

Tenants in SROs, who grapple with unemployment, poverty, racism, and mental and physical health challenges, often experience various forms of interpersonal and structural stigma that diminish their ability to exercise agency. SRO staff and fire inspectors frequently perceived that residents in the DTES were unwilling to follow the rules. However, some staff acknowledged that tenants’ mental health challenges made it more difficult to enforce fire safety practices.

During a false alarm in an SRO building, a local firefighter expressed concerns about the lack of compliance with fire safety regulations, noting that “policymakers sometimes fail to consider the mental health issues faced by SRO tenants.” Staff at an SRO building directly across from a petrol station reported that tenants covered smoke detectors due to frequent alarm sounds and smoking in their rooms. However, one tenant claimed he covered his smoke detector because he believed fumes from the nearby gas station frequently triggered the alarm, contradicting the staff’s perspectives. A private SRO owner expressed frustration after receiving a violation notice for breaching fire safety bylaws due to poor building conditions. He lamented, “You give rooms to people from the streets, and this is the problem we see in these buildings,” indicating his perception that tenants were responsible for damaging the building.

During a hoarding reinspection that resulted in a “do not occupy” notice on a tenant’s door, staff observed, “This tenant uses the room as a storage space and sleeps out in the street.” Staff from various SROs reported that many tenants preferred living on the streets and using their rooms for storage because “the streets did not have any rules.” They expressed concerns that individuals with co-morbid mental health and substance use challenges were putting others at risk by not following rules and engaging in “risky behaviours.” On another visit, a long-term tenant followed the inspection team and remarked, “I have lived in this building for 22 years, and lately, junkies who have moved in have deliberately set off the fire alarms—such a nuisance.” Some staff acknowledged their views might be controversial, but they believed having SROs in Vancouver’s downtown, considered a “prime location,” was problematic. They expressed confusion about why SROs remain in this area and suggested that the government relocate SROs out of the city, as their presence negatively impacts tourism and the economy.

Some staff expressed frustration with the daily challenges of their job, remarking that “people who live in these SROs are unfit to live independently.” Others were frustrated with the so-called “soft culture” towards people with mental health challenges, arguing that merely “meeting individuals where they are at” instead of “institutionalizing them” represents a systemic failure. This reference to institutionalization pertains to the closure of the Riverview Mental Health Hospital, located approximately 26 km from DTES, which served thousands of patients with mental illness until its closure in 2012. This closure aimed to reintegrate individuals with mental health issues back into their communities [65]. Staff felt that community-based mental health services were already overburdened and did not adequately meet the needs of people in DTES.

SRO staff and firefighters conveyed feelings of “compassion fatigue” as they grappled with a sense of helplessness while working with individuals who use “drugs” and those experiencing “mental health problems.” One staff member remarked, “Higher-ups do not have a clue what it is like on the ground.” Another staff member expressed frustration, questioning the need for strict regulations, given that the residents of supportive housing generally have co-morbid mental health challenges. They stated, “You have housing like this [SROs]; they [housing association] know that people who live in supportive housing have mental health issues, and 90% of them are using drugs, so why do we have so many restrictions?”

## Discussion

The findings of this study have deepened our understanding of the importance of identifying contextual factors that contribute to urban fire hazards for individuals inadequately housed in SROs. Specifically, the impact of stigma on people with co-morbid challenges of substance use and mental health has potentially shaped the narratives surrounding fire events in underserved communities. Our findings demonstrate that structural factors are commonly overlooked in fire and burns research. Although five themes have been discussed in this study, collectively, they highlight structural factors generated by multiple, interlocking systems of oppression (such as discriminatory practices and systems that work together to marginalize individuals).

The structural deficiencies in SRO-type housing create a marginal, segregated, and harmful living environment. Additionally, SRO tenants who are socially and materially disadvantaged face intersecting stigmas related to substance use and poverty. These stigmas can amplify the existing marginalization experienced by these individuals, further exacerbating their health and social inequities [33, 56]. Individuals living in permanent supportive housing are characterized as having high-risk behaviours and challenges due to mental health and substance use issues, including histories of homelessness [66]. However, this focus on individual attributes diverts attention from the lack of services provided to those with comorbid challenges in permanent supportive housing. Although SROs are preferred over shelter accommodation and have addressed contemporary homelessness [42], the commodification and financialization of housing within these SROs have increased individuals’ vulnerability and susceptibility to adverse health outcomes due to stressors associated with fire hazards and fires. In contrast, residents in wealthier neighbouring areas and refurbished heritage buildings within DTES face significantly lower fire risks, thus experiencing fewer fire-related stressors.

Hoarding issues in supportive housing are recognized as significant fire risks [66]. However, hoarding is often framed as an individual behavioural issue resulting from traumatic life experiences, such as intimate partner violence, childhood abuse, and violent victimization, as well as mental health challenges, cognitive impairments, and difficulties with daily activities [67, 68]. The significance of structural-level factors, such as excessive regulation, coercive environments, inadequate space, and inappropriate living conditions in permanent supportive housing, is often overlooked in the literature on hoarding. Insufficient space is a significant housing stressor that adversely affects tenants’ mental health in SROs [42]. Although hoarding is described as a complex mental health issue, our findings indicate that it is further exacerbated by the limited resources available to municipal services for addressing and preventing hoarding [69].

Hoarding assessments for SRO tenants facing eviction due to fire risks often fail to consider contextual and structural factors. The Clutter Image Rating (CIR) assessment, designed to evaluate the degree of clutter in an SRO room of 100 square feet or smaller, is both challenging and overly reductionist in its methodology. Imposing punitive measures, such as forcing tenants to dispose of their belongings or face eviction, exacerbates homelessness and intensifies stressors, leading to increased risks of mental health crises. These reductionist approaches exemplify how poverty and social insecurity are increasingly criminalized [70]. CIR reflects broader societal changes under neoliberalism, where individual responsibility and self-management are emphasized, and assessment tools are used for managing risks rather than addressing dangerousness [71].

Although the Hoarding Action Response Team (HART) approach has proven effective in supporting individuals exhibiting hoarding behaviour outside the DTES area [72], it has not been implemented in DTES. Our findings support the literature that community members benefit more from an interdisciplinary and community-centred approach that involves tenants in decision-making processes and incorporates harm-reduction strategies [72]. Instead, tenants in SROs who accumulate possessions due to inadequate storage facilities are often subjected to coercive decluttering procedures administered under the threat of eviction. These differing approaches to managing hoarding highlight how negative labelling and stereotyping of geographic areas and their residents result in reduced access to services, social exclusion, and further marginalization [73].

Participant observation in this study revealed that fire safety education primarily caters to populations with digital accessibility and those capable of exercising agency, often neglecting individuals facing societal stigmas and significant barriers. Fire and emergency services cover various educational topics but focus mainly on lifestyle behavioural changes [74]. Furthermore, empirical studies on fires and burn injuries heavily rely on statistical data, which shapes the design of prevention and intervention programs.

Individuals who use drugs and reside in SROs are often unfairly stigmatized as violent, immoral, and criminal [35]. Participant observations highlighted that fires and hoarding issues, attributed to high-risk behaviours, frequently lead to the victimization of tenants. These narratives create barriers for individuals to assert their agency as they contend with ongoing dehumanization. When individuals attempt to exercise their agency by challenging coercive practices related to the City’s fire bylaws, they are often labelled non-compliant and deemed “problematic” (individual behaviour).

Stigma is a fundamental cause of population health inequities and is enacted through policies, laws, and programming at the structural level [56]. The different approaches to managing fire safety and risks are evident in neighbourhoods undergoing state-sponsored gentrification through socially mixed development [73], such as Vancouver’s DTES. Moreover, the perception that DTES residents are somehow “less than us,” influenced by neoliberal and capitalist ideologies, suggests that their perceived lack of contribution through taxes denies them access to essential community infrastructure like housing and services. The government’s role in perpetuating or alleviating housing and poverty issues is primarily hidden due to poverty propaganda, which orchestrates confusion and misunderstanding [33]. Emphasizing individual-level factors in fire events among social housing residents is misleading because it obscures the realities of the government’s role in promoting fire safety in underserved communities. The stigmatizing narrative about these fire events in an underserved community becomes justifiable because of the perceived belief that individuals’ careless and irresponsible behaviour causes these fires [33].

Despite having a significant Indigenous population, fire safety programs implemented in the DTES neighbourhoods lack considerations of cultural safety, human rights, and justice [75–77]. In Canada, the prevalence of homelessness among Indigenous Peoples is eight times greater than that among non-Indigenous individuals [62]. Challenges related to substance use, mental health struggles, and homelessness among Indigenous Peoples stem directly from structural inequities and trauma linked to colonization [61]. Stigma and discrimination against Indigenous identity function as social determinants of health, affecting those living in structurally disadvantaged neighbourhoods [78, 79].

### Limitations

This analysis of participant observation data from an ongoing study has several limitations. The research was conducted in a unique setting and may not reflect the experiences of individuals inadequately housed in SROs in other parts of Vancouver or elsewhere. The analysis draws on data generated through participant observations, policy analysis, and conversations with individuals living and working in DTES. Additional interviews, which the first author is currently conducting, would be beneficial to deepen the analysis. Despite these limitations, the following section highlights some of the key implications.

### Implications for practice

Housing is a physical structure and a network of social relations. While access to SRO housing is crucial for addressing homelessness, individuals living in SROs often face undesirable physical and social conditions that adversely affect their health and well-being, including increased fire risks and hazards. Fire and burn injuries are public health concerns, and therefore, all elements contributing to increased fire hazards—such as stigma affecting those inadequately housed in SROs—must be considered social determinants of health and addressed through principles of equity.

An equity-oriented approach to fire safety in underserved communities encourages an analysis of fire hazards and risks from a structural lens and not solely at an individual level. This approach requires a contextual understanding of how individuals in underserved communities navigate their surroundings, obtain housing, and address fire safety concerns. This understanding is essential for creating equitable responses to fire risks and safety programming, such as providing adequate housing and ensuring people’s safety.

While comprehending the etiology of hoarding is crucial, recognizing how structural violence has influenced and shaped the traumas experienced by individuals displaying hoarding behaviours is equally vital. Municipal staff working in these areas should have the tools, resources, and education to approach hoarding with a trauma-informed and violence-informed care approach (TVIC). A TVIC approach directs our focus towards the broader societal factors influencing individuals’ health, encompassing persistent forms of violence such as structural, systemic, and institutional, alongside discrimination and detrimental practices ingrained within both systems and societal norms [80]. Assessments of hoarding in SROs should encompass beyond merely guaranteeing unobstructed hallways and room entrances for fire safety. They should evaluate individuals’ capacity to engage in vital daily tasks and flourish within their housing environments.

Developing contextual programs incorporating the insights of people most impacted by fire hazards in DTES is essential for adequate fire prevention and safety measures. While it may appear more practical to attribute blame to individuals and implement targeted interventions to modify behaviours, such as regulating the use of “smokers’ material” or “retraining” people to dispose of garbage, the underlying structural issues persist. Tailored interventions, though costlier, are outweighed by the expenses from fire calls and healthcare for burn injuries. Social and structural inequities will continue to persist if upstream efforts are not considered, such as providing adequate housing and attending to stigmatizing attitudes, thus leading to the perpetuation of structural violence [81].

Finally, a comprehensive analysis of fire risks, prevention, and safety must consider historical and sociopolitical contexts. Addressing the pervasive and negative impacts of systemic and interpersonal racism is essential. Providers and organizations must recognize their roles and responsibilities in tackling these issues [82]. Adequate fire safety interventions should include strategies to counteract intergenerational trauma by engaging with Indigenous elders and partners. This approach incorporates Indigenous knowledge and perspectives into fire risk and safety programming, including the social and physical housing environments.

## Conclusions

Fires in underserved communities highlight broader systemic deficiencies, where inadequate focus on structural factors such as housing availability, income adequacy, and the absence of intersectoral collaboration has perpetuated a narrative of individual blame. Mitigating these risks should include enhancing collaboration among fire, health, and sanitation programs. Additionally, educating staff on anti-bias and trauma- and violence-informed care (TVIC) approaches is essential to serve better communities experiencing social exclusion, racism, stigma, and inequities. Contextualizing fire risks and hazards in underserved communities will improve our understanding of the structural factors that create these risks in the first place. Recognizing these interconnections allows us to move beyond modifying individual behaviours to addressing the underlying causes of fire risks in underserved communities.

### Electronic supplementary material

Below is the link to the electronic supplementary material.

Supplementary Material 1

## Data Availability

The datasets generated and/or analyzed during the current study are not publicly available due to anonymity requirements. We are still actively working on analyses, but they are available from the corresponding author upon reasonable request.

## Footnotes

1 In this paper, the authors define adequate housing as encompassing physical, spatial, psychological, emotional, and social dimensions.

2 Seventy-two people died, and many were displaced from their homes in the fire at Grenfell Tower, a council-owned high-rise social housing block in the London Borough of Kensington and Chelsea, an affluent neighbourhood in London, UK [31].

3 The fire at the Winters Hotel (social housing) displaced more than 70 residents and killed two. The Winters Hotel is a heritage building in Gastown, a retail and commercial district in Vancouver. It is classified as an SRO and operated by a charitable organization funded by BC Housing [32].

4 Visible minority is a term defined by the Government of Canada for populations who do not have characteristics like the dominant population (people of European ancestry) as visible minority: “persons, other than Aboriginal peoples, who are non-Caucasian in race or non-white in colour” [45].

5 Approval from the FNHA was sought due to the study’s engagement with First Nations communities and individuals. Although data collection is ongoing for this aspect of the study, the findings are not included in this paper.

6 The Braun and Clark’s [42] six steps approach is: (1) deepening familiarity with the dataset, (2) generating the early codes, (3) generating initial themes, (4) reviewing the themes, (5) refining, defining and naming themes, and (6) writing up the report.

7 Stigma as a result of discrimination towards people who use drugs [56].

8 Stigma reflected in policies and practices that lead to unfair standards and treatments [57].

9 Naloxone is a fast-acting medication used to temporarily reverse the effects of opioid overdose (fentanyl, heroin, morphine, and codeine) by restoring breathing within 2–5 min [58].

10 Since the Public Health Emergency was declared in BC on April 14, 2016, there have been 13,112 recorded deaths due to drug toxicity up to November 1, 2023 [59].

11 These sentiments were fueled by a 2023 incident, wherein a scathing report revealed a culture of conflict of interest, with the husband, CEO of BC Housing, awarding housing contracts and projects to his wife, the CEO of the largest non-profit housing provider [63].

12 A “do not occupy” notification is categorized as a public safety (activity category) informing the tenants that occupying is unsafe. It is a legal notice issued by the VFRS and issued when the premise does not conform to the Vancouver Fire By-Law.

## Abbreviations

DTES: Downtown Eastside
SRO: Single room occupancy
BC: British Columbia
VFRS: Vancouver Fire Rescue Services
CE: Critical Ethnography
UCT: Urban Compliance Team
HART: Hoarding Action Response Team
CEO: Chief Executive Officer
CIR: Clutter Image Rating
TVIC: Trauma and Violence Informed Care
