# Supplementary material for: Beyond individual factors: a critical ethnographic account of urban residential fire risks, experiences, and responses in single-room occupancy (SRO) housing
Source: BMC Public Health. 2024 Aug 28;24:2343. doi: 10.1186/s12889-024-19866-z (PMC11360511; doi:10.1186/s12889-024-19866-z)
Supplement: Supplementary file 1 — Supplementary Material 1 [file 12889_2024_19866_MOESM1_ESM.pdf]

Field Note Template

Date: **Time:** **Site:**

Participant Observation

Setting Observations:
- Describe the physical location and spatial characteristics of the community.
- Observe and note the surrounding environment and its impact on the community.
- Document the characteristics and diversity of community members residing in this environment.
- Explore the neighbourhood dynamics and any observable social interactions.

Access to Social Determinants of Health:
- Observe and document the condition of housing and other buildings in the community.
- Assess the accessibility and quality of roads and transportation options available.
- Evaluate the community's access to water sources, including fire hydrants.
- Note the availability and adequacy of sanitation facilities and hygiene needs.
- Investigate the accessibility and utilization of health and social services by community members.

Decision-Making Processes:
- During community meetings and interactions with decision-makers and service providers, engage in stationary observation.
- Observe and document how decisions are made within the community.
- Pay attention to the involvement and participation of community members in decision-making processes.
- Analyze the capacity of community members for self-decision-making and their influence in shaping decisions.

Community-Level Communications:
- Observe and record the various forms of communication within the community, such as signs, ordinances, and policies.
- Examine how institutional power is communicated through signage, memos, professional identities (uniforms), bylaw posters, and community notices.
- Analyze the messages these communication methods convey and their impact on community members.

Interactions Among Community Members and Leaders/Service Providers:

- Observe and document the interactions between community members and community leaders or service providers.
- Analyze the power dynamics and systemic oppressions that may manifest during these interactions.
- Note any variations such as interactions based on factors like social status, Indigeneity, ethnicity, or gender.

Reflections on Researcher Positioning:

- Reflect on my thoughts, emotions, and experiences as a researcher.
- Analyze how my presence and visibility may be influencing the dynamics of the observed community.
- Consider the evolving relationship between myself and community members or leaders and how it affects my observations.

Identify Patterns and Themes; Ask Questions:

- Identify any recurring patterns or themes from the participant observations.
- Reflect on the significance of these patterns and their implications.
- Ask relevant research questions based on the observed patterns and identify gaps.

Sample Interview Guide for Burn Survivors

1. **Preamble:** As you know, I am interested in learning about people's experiences of burn risks and care, and what can be done to improve those experiences. Where would you like to start? (alternatives depending on the response)Would you be comfortable starting by telling me what led to your burn injury?

Follow up as needed:
Immediate response and actions (personal experience)

a. What were the circumstances or factors that contributed to your burn injury?
b. What immediate steps did you take to address the burns or minimize its impact?

Treatment and Care Journey

c. Can you share your experience of receiving treatment and care following the injury?
d. What medical interventions or therapies were involved?
e. How did you handle the healthcare system, and what difficulties or positive experiences did you encounter while navigating it?

Recovery Process

f. Could you describe your recovery process after the burn injury?
g. What kinds of support or resources were available to you during your recovery?
h. Did you feel adequately supported in terms of physical, emotional, or financial assistance? Were there any gaps or limitations in the support you received?
i. What were the challenges or barriers encountered in accessing appropriate care?

Impact on various aspects of life

j. How did the burn injury and subsequent recovery impact various aspects of your life, such as personal relationships, social interactions, or daily activities?
k. Can you discuss any challenges or changes you experienced in these areas?

Risks

l. What are some common risks and hazards related to burns that you have noticed or experienced in your everyday life or community?
m. Are there any specific cultural beliefs, customs, or behaviours that influence how people think about the dangers of fire or burns (risks)?

[**Research Question:** How do underserved populations experience burn injury and understand risks?]
Purpose: To gather information about unique/personal stories from burns survivors- how and why did they sustain their injuries]

2a. Preamble: I am now interested in learning more about how certain factors related to your living environment could have played a role in causing your injury

a. Considering your living environment, what aspects or factors do you believe played a role in your burn injury?

b. Can you provide more details about any specific things in your community or surroundings that might have made it riskier in terms of potential danger, conditions, hazards, or challenges related to burns?

2.b Preamble: Now can you tell me if the opinions and beliefs of society affect how you asked for or received help with the burn injury?

Follow-up questions:

a. Have you noticed how people in society generally view or think about individuals who have suffered burn injuries in your community?
b. Can you describe any instances where you felt unsupported or stigmatized?
c. Did you experience any unfair treatment, unequal opportunities, or lack of sufficient support while you were recovering?
d. Did you encounter any specific obstacles or difficulties in getting the assistance you required? These barriers could be related to systems or structures that made it more challenging for you to access the help you needed.

[Research Question: How do contextual factors such as social, cultural, political, historical, and economic factors influence burns risks and care experiences?
[Purpose: To gather information about the barriers and facilitators that influences burns risks and care experiences]

2. **Preamble:** Recovery from burns can be a long journey. I am interested in learning more about your social network and support, and what role did it play in your recovery.
a. How did your social networks, such as family, friends, or community members help you in providing emotional support or practical assistance?
b. Were there any specific organizations, community programs, or support groups that provided assistance or guidance?
c. Can you share if your situation and experience affected how you seek help or support?
d. Can you describe if there were any instances you felt unsupported?

[**Research Question:** How do underserved populations utilize social support to cope with burn injuries?]
[Purpose: To gather information about how underserved populations seek formal and informal to cope with burn injuries]

3. **Preamble:** I would like to know your thoughts on how the support available for you and others in your community with burn injuries could be improved. What recommendations or changes would you suggest to enhance the existing support systems?

Follow-up questions

a. Are there any existing policies, programs, or initiatives in place that address burn prevention and care within your community or broader society?
b. Are there any specific recommendations or initiatives that you believe would have a significant impact?

[Research Question: What services or strategies could reduce burn risks and optimize burn care for underserved populations- insights from burn survivors?]
[Purpose: To gather information about the gaps in burns prevention, treatment, and recovery; and identify collective solutions].

4. Is there anything you would like to add?

Sample Interview Guide for Community Leaders & Service Providers\

Roles and Experiences/Organizational Support/Contextual Factors

1. **Preamble:** As you know, I am interested in learning about inequities in burns risks, and care, what shapes those inequities, and what can be done to reduce them. Can you tell me about your role and experiences with burns?

[Possible follow-up as needed]

a. How do you think the community sees your role in addressing fire and burns risks and care?
b. What factors influence how the community perceives you and your role?
c. How has your organization supported your role in addressing fire and burns risk, safety, prevention, or care?
d. Can you describe the resources, policies, or initiatives that have helped fulfill your responsibilities?
e. What factors have influenced the support you have received from your organization?
f. What factors influence the risk of burn injuries in the community you serve?
g. Can you identify any factors impacting an individual's experiences accessing fire or burns safety, prevention, and care?

[**Research Question:** How do contextual factors such as social, cultural, political, historical, and economic factors influence burns risks and care experiences?]
Purpose: To gather information about the barriers and facilitators that influences burns risks and care experiences]

Every day Burn Injuries/Impacts/Experiences and Addressing Inequities

2. **Preamble:** Now, I am interested in learning about the common burn injuries you frequently see in the community where you work and serve.

Follow-up questions

a) Can you explain why these specific types of burn injuries are more common?
b) Can you explain if there are structural factors that contribute to these patterns?
c) Are there interventions or programs in the community to reduce burn injuries?
d) Can you describe these interventions and their impact on mitigating fire/burns risks?
e) What were the reasons for their success or lack of effectiveness?
f) Can you share your experiences with burn survivors in the community?
g) Have you observed inequities in the care experiences of the community where you serve?
h) What common challenges or needs do burn survivors face in accessing health and social care?
i) Do you know how burn injuries affect their physical, emotional, and social well-being?

[**Research Question:** How do underserved populations understand burns risks and care?]
[**Purpose**: To gather information from the perspectives of community leaders about how and why underserved populations are at higher risks of burn injury and what might their care experience be?]

Community Awareness and Support

3. **Preamble:** I am interested in learning more about how you ensure the community is aware of the services you provide or your role.

Follow-up

a. What strategies or initiatives do you undertake to raise awareness among the community members?
b. What factors might prevent individuals in these communities from accessing your services or other available services?
c. Are the barriers or challenges hindering access? (e.g., financial constraints, lack of information, cultural beliefs, or systemic issues).

[**Research Question:** How do underserved populations utilize health care services and social support to cope with burn injuries?]
[**Purpose:** To gather information from community leaders on whether they help underserved populations navigate the health and social system].

Support and Services/Gaps and Collaborative efforts

4. **Preamble:** Now, I am curious to learn what support and services there are to reduce burns risks and optimize care in your community. Can you provide an overview of the resources, programs or initiatives available in the community to minimize fire/burn injury risks and optimize care?

- Do these support and services effectively address burn risk and optimize care? If yes, why? If not, what are the reasons for their lack of effectiveness?
- How do you believe fire and burns risks can be reduced and care optimized in the community?
- Can you identify gaps, challenges, limitations, or unmet needs in the current support and service?
- Are there any collaborative efforts you suggest to enhance burn prevention, treatment, and recovery in the community where you serve?

[Research Question: What services or strategies could reduce burn risks and optimize burn care for underserved populations?
[Purpose: To gather information about the gaps in burns prevention, treatment, and recovery; and identify collective solutions].

5. **Is there anything you would like to add?**
